# Effect of strontium ranelate on pain behavior in an experimental model of osteoarthritis

**DOI:** 10.1590/1414-431X20176314

**Published:** 2017-08-07

**Authors:** T.A. Rodrigues, A.J.B. Sampaio, I.D.P. Nunes, M.S.S. Cartágenes, J.B.S. Garcia

**Affiliations:** Centro de Ciências Biológicas e da Saúde, Universidade Federal do Maranhão, São Luís, MA, Brasil

**Keywords:** Osteoarthritis, Strontium ranelate, Pain, Treatment, Prophylaxis

## Abstract

Strontium ranelate (SrRan) is a drug usually prescribed to treat osteoporosis, with proven effects of decreasing the risk of fractures and an indication of reducing the progression of osteoarthritis (OA). This study aimed to investigate the effects of SrRan as either a prophylactic or a treatment drug, using an OA rat model to assess pain behavior. A monoiodoacetate (MIA)-induced knee joint OA model in Wistar rats was used. Thirty Wistar rats (both sexes, 60 days old) were distributed in five groups of 6 rats each: the control group, that received no intervention; a prophylactic group, that received oral administration of 25 mg·kg^-1^·day^-1^ of SrRan for 28 days before induction of OA; a group treated with 25 mg·kg^-1^·day^-1^ of SrRan for 28 days after OA induction; a group treated with 50 mg·kg^-1^·day^-1^ during 28 days after OA induction; and a group that received oral saline for 28 days after induction. The assessment of pain behavior was performed considering articular incapacitation (weight-bearing test), mechanical hyperalgesia (Randall Selitto test) and motor activity (rotarod test), on days 0, 7, 14, 21, and 28. This experiment did not yield a significant difference when comparing the group that received SrRan prophylactically with the groups treated with 25 or 50 mg·kg^-1^·day^-1^ and the group that received oral saline. Thus, SrRan did not provide analgesia in either treated rats or as a prophylactic drug with the tested doses. Higher doses should be tested further to achieve possible significant results.

## Introduction

Advances in the understanding of the pathophysiology of osteoarthritis (OA), such as the influence of biochemical stress or abnormal intra-articular biomechanics, and the inflammatory pathways involved, have allowed for a considerable increase in therapeutic targets for the disease. Some medications have been associated with reduction of cartilaginous lesions and decreased subchondral bone remodeling, changing the progression of OA (1–5). These drugs, known as disease-modifying osteoarthritis drugs (DMOADs), present the properties of reversing, stabilizing, or at least delaying the course of OA. Oral DMOADs of note include chondroitin (6), diacerein (7), glucosamine (8), glucosamine combined with chondroitin (9), and chloroquine (10). Among the intra-articular treatments, viscosupplementation with hyaluronic acid (11) is also worth highlighting.

Studies have been conducted to increase the number of medications that effectively reduce the progression of OA. The drug strontium ranelate has shown promising results in the prevention of fractures and treatment of osteoporosis in postmenopausal women (12–14), indicating its probable utility in the treatment in OA (2 [Bibr B03] [Bibr B04],^15^-^17^).

Strontium (Sr, group II of the periodic table with atomic number = 38) is a fundamental element. Its nucleus is similar in size to calcium, making it easily absorbed, carried, and incorporated into bones, as is calcium (15). Its ability to decrease pain and increase bone density was first assessed radiographically in 1959, in a small study including patients with osteoporosis treated with strontium lactate (18). Thus, it has already been used in the treatment of osteoporosis for decades (19).

Currently, strontium ranelate (SrRan - C_12_H_6_N_2_O_8_SSr_2_) is indicated for the treatment of severe OA and osteoporosis, especially in postmenopausal women with a high risk of fractures (16,19–21). It has also been postulated that SrRan inhibits the resorptive activity of osteoblasts, thus reducing the synthesis of metalloproteinase. In addition, it modulates the osteoprotegerin-RANKL (receptor activator of nuclear factor kappa-B ligand) signaling pathway, and inhibits osteoclastic differentiation (15). SrRan has also been associated with the formation of cartilaginous matrix. Recent data show that it can reduce the progression of radiological findings in spinal OA, along with leading to improvement of lower back pain in women with osteoporosis (15).

Although SrRan shows the ability to reduce the progression of OA, few relevant studies have been published to date. Therefore, the present study aimed to test the effects of SrRan in an experimental animal model of OA induced by intra-articular injection of sodium monoiodoacetate (MIA). Clinical assessment of motor activity, articular incapacitation, and mechanical hyperalgesia was conducted.

## Material and Methods

This study was conducted in the Experimental Laboratory for Pain Study (LEED) following approval from the Animal Ethics Committee of the Universidade Federal do Maranhão (CEUA-UFMA No. 23115.012456/2016-4).

### Animals

Thirty Wistar rats, *Rattus norvegicus* species (albino variety), were used in the study. The animals were male and female adults, approximately 60 days old. This study did not aim to evaluate differences between genders. The rats were obtained from the Central Animal Facility of the Universidade Federal do Maranhão. The animals remained in cages and were housed at the LEED lab, where they were fed standard chow and water *ad libitum* and maintained under controlled conditions of light and temperature.

### Experimental design

The animals were divided into five groups (PROF25, SR25, SR50, SAL, and Control), with 6 rats each. Group PROF25 (prophylactic group) began treatment with SrRan 4 weeks prior to the induction of OA with sodium MIA. SrRan was administered in a dose of 25 mg/kg by gavage, once daily in the morning, two hours before the subsequent feeding. Groups SR25 and SR50 (treatment groups) received 25 mg/kg and 50 mg/kg of SrRan, respectively, starting from day 7 after OA induction, by gavage, once daily in the morning, 2 h before subsequent feeding, for a period of 4 weeks. Group SAL received 0.9% saline solution by gavage after OA induction. The day of OA induction in groups PROF25, SR25, SR50 and SAL was considered day zero (D-0). The Control group did not undergo OA induction nor received intervention. The administered doses in that model were chosen based on previously described animal models using SrRan (15). Throughout the experiment, all groups were periodically evaluated for articular incapacitation, motor activity, and mechanical hyperalgesia on days 0, 7, 14, 21, and 28, as described below.

### Model of osteoarthritis induced by sodium monoiodoacetate

To induce OA, the animals were anesthetized using an intraperitoneal injection of 40 mg of sodium thiopental. The joint injury was induced with a single intra-articular injection of 2 mg of sodium MIA into the right knee, diluted in a maximum volume of 25 µL solution (22,23).

### Evaluation of motor activity - forced ambulation (rotarod test)

The animals were placed on a rotarod (IITC Life Science, USA) at a speed of 16 rpm for a period of 300 s. The use of the affected limb was evaluated through forced ambulation. The use of the affected paw was graded by a subjective measure, on a numerical scale ranging from 5 to 1, in which: 5=normal use of the paw; 4=mild limping; 3=severe limping; 2=intermittent disuse of the affected paw; 1=complete disuse of the affected paw (24).

### Incapacitation test - distribution of weight on the hindpaws (weight bearing test)

The animals were placed in a glass chamber, angled and positioned so that each hindpaw rested on a different platform. The weight exerted on each hindpaw (measured in grams) was evaluated over a period of 5 s. The final weight distribution was calculated using the average of three measurements. The variations in distribution of the weight on the hindpaws were calculated using the following formula:

$$\text{Weight distribution (\%) =}\frac{APW}{APW+CPW}\times 100$$

where APW was affected paw weight and CPW was contralateral paw weight.

### Mechanical hyperalgesia (Randall Selitto test)

The Randall Selitto test is a useful test to evaluate the hypernociception, based on the induction of hyperalgesia by the increasing paw pressure using a special device. Mechanical hyperalgesia was assessed using the nociceptive paw-withdrawal threshold (NPWT) to mechanical pressure using an analgesy-meter (IITC Life Science) (25,26) in both paws, and then calculating the mean of three measures. The paw withdrawal reflex is considered representative of the hypernociceptive threshold. The NPWT was recorded in grams for both paws, and then the percentage was calculated using the results of affected and contralateral paws, with the following formula:

$$\text{NPWT (\%) =}\frac{NAPWT}{NAPWT+NCPWT}\times 100$$

where NAPWT is nociceptive affected paw-withdrawal threshold and NCPWT is nociceptive contralateral paw-withdrawal threshold.

### Statistical analysis

Comparison of the means of different experimental groups was performed using the Student’s *t*-test or univariate analysis of variance (one-way ANOVA), followed by the Bonferroni’s test. A value of P<0.05 was considered indicative of significance and the data obtained were analyzed using the GraphPad Prism® software, version 6.00 for Windows (USA).

## Results

### Evaluation of articular incapacitation

**Figure 1.**
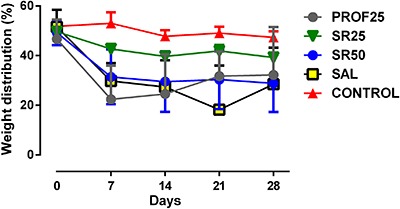
Evaluation of articular incapacitation using the weight bearing test. Group PROF25: administration of prophylactic 25 mg/kg of strontium ranelate (SrRan) 4 weeks prior to the induction of monoiodoacetate (MIA)-induced osteoarthritis (OA); Groups SR25 and SR50 (treatment groups) received 25 mg/kg and 50 mg/kg of SrRan, respectively, after OA induction for a period of 4 weeks; SAL: MIA-induced OA receiving only saline; Control group: no treatment and no OA induction. Results are reported as means±SD. P<0.05 between groups PROF25, SR25 and SR50, and Control; P>0.05 between those groups and SAL (one-way ANOVA, followed by the Bonferroni’s test).

The analysis of data regarding articular incapacitation, assessed using the weight bearing test, demonstrated that the OA induction was effective, as we observed a statistically significant difference between the OA group that received saline (group SAL) and Control group (healthy animals that received saline). A statistically significant difference was observed between the PROF25 (prophylactic) group and the Control group (P<0.05). However, no difference was observed between groups PROF25 and SAL. These results showed that the animals that received SrRan prior to OA induction did not approach the healthy standard of the Control group, nor were they significantly different from those that received saline (Figure 1).

We observed a difference between the groups that received 25 and 50 mg/kg SrRan (SR25 and SR50), and the group of healthy animals (Control). These groups did not significantly differ from group SAL (Figure 1).

### Evaluation of motor activity/forced ambulation

**Figure 2.**
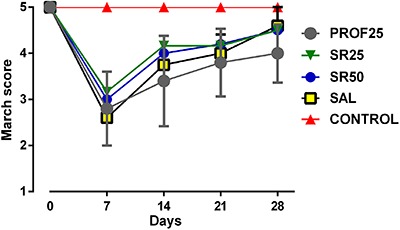
Evaluation of motor activity/forced ambulation using the rotarod test. Group PROF25: administration of prophylactic 25 mg/kg of strontium ranelate (SrRan) 4 weeks prior to the induction of monoiodoacetate (MIA)-induced osteoarthritis (OA); Groups SR25 and SR50 (treatment groups) received 25 mg/kg and 50 mg/kg of SrRan, respectively, after OA induction for a period of 4 weeks; SAL: MIA-induced OA receiving only saline; Control group: no treatment and no OA induction. Results are reported as means±SD. P<0.05 between groups PROF25, SR25 and SR50, and Control; P>0.05 between those groups and SAL (one-way ANOVA, followed by the Bonferroni’s test).

The rotarod test showed that the OA induction was effective, resulting in a statistically significant difference between groups SAL and Control. We also observed a statistically significant difference between the PROF25 and the Control groups. The same difference was found between the groups that received treatment with SrRan (SR25 and SR50) and the Control group. A difference was not observed between groups PROF25 and SAL, SR25 and SAL, or SR50 and SAL. These findings indicated that there was no change between the animals receiving SrRan and those receiving only saline (Figure 2).

### Evaluation of mechanical hyperalgesia

**Figure 3.**
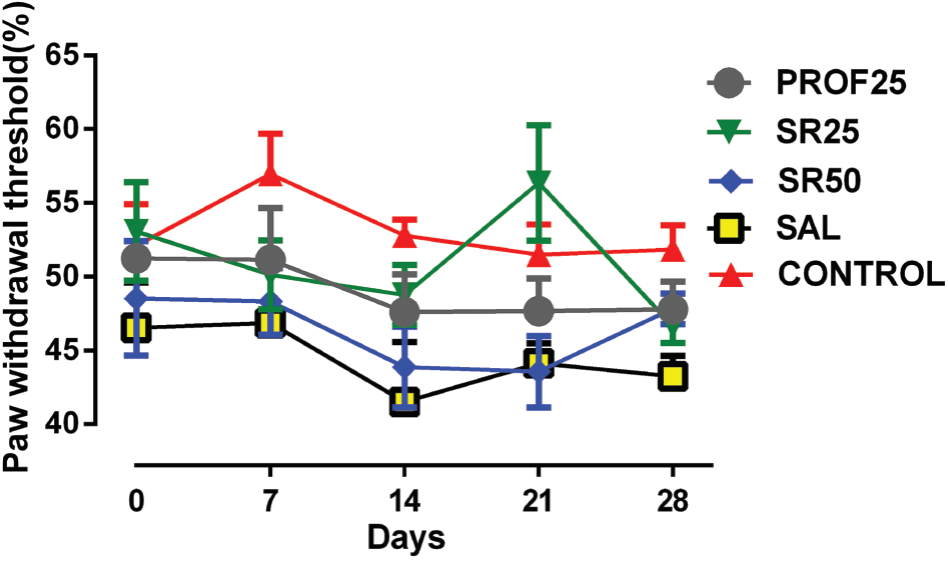
Evaluation of mechanical hyperalgesia using the Randall Selitto test. Group PROF25: administration of prophylactic 25 mg/kg of strontium ranelate (SrRan) 4 weeks prior to the induction of monoiodoacetate (MIA)-induced osteoarthritis (OA); Groups SR25 and SR50 (treatment groups) received 25 mg/kg and 50 mg/kg of SrRan, respectively, after OA induction for a period of 4 weeks; SAL: MIA-induced OA receiving only saline; Control group: no treatment and no OA induction. Results are reported as means±SD. P<0.05 between groups PROF25, SR25 and SR50, and Control; P>0.05 between those groups and SAL (one-way ANOVA, followed by the Bonferroni’s test).

Evaluation of hyperalgesia by means of the Randall Selitto test also showed that the OA induction was effective, with a statistically significant difference observed between groups SAL and Control. A statistically significant difference was also found with this test between the prophylactic group and the healthy group. In addition, a difference was observed when comparing the groups treated with SrRan (SR25 and SR50) and the group consisting of healthy animals (Control). We did not observe any difference between the prophylactic group, groups receiving treatment, and group SAL (Figure 3).

## Discussion

The present study assessed pain behavior in an experimental model of OA, using a drug that has been studied as a potential pharmaceutical to be included in the class of DMOADs.

At the administered doses and with the tests employed, there was no observed improvement with the use of SrRan in cases of established OA. This outcome differs from the findings of another study in which OA was induced in rats by zymosan. In that case, the animals were treated with higher doses of SrRan than in our study, ranging from 30 to 300 mg/kg^–1^·day^–1^ for a shorter period of time (27). The difference could be probably attributed to the different model applied, with higher doses used in that study.

The choice of the SrRan doses administered in our study was based on a study with dogs that underwent OA induced by the section of the anterior cruciate ligament. The doses used were 25, 50, or 75 mg/kg for a longer period (12 weeks, beginning 4 weeks after the surgery) than in our study (15). That study was the first to demonstrate *in vivo* the effect of SrRan in reducing the OA progression. The main differences between our study and the above one were the OA induction method and the duration of the treatment. Such differences could have influenced the varying results between the studies.

The prophylactic effect of SrRan was not shown in this experimental study using a model of MIA-induced OA. We did not observe a reduction in articular incapacitation after OA induction, nor was there an improvement in the motor response in the group that received 25 mg/kg SrRan for 1 month prior to OA induction. Otherwise, the prolonged prophylactic use of SrRan has already been shown to be associated with a reduced fracture risk in some clinical trials, especially in postmenopausal women with osteoporosis (13,21). It has been suggested that this prophylactic effect is due to an anti-resorptive and pro-formation action in bone metabolism processes (14,21).

The exact mechanism of action of the substance is not entirely clear (17). However, the regulation of bone cell differentiation, the stimulation of osteoblast proliferation, and the inhibition of osteoclast formation with probable apoptosis of "mature" cells have been suggested as possible mechanisms, in addition to the activation of receptors sensitive to calcium – the latter being the most probable (17,19,28–30). It has also been shown *in vitro* that SrRan increases the synthesis of collagen and non-collagenous proteins, improves the proliferation of pre-osteoblast cells, and that it should, therefore, be classified as a bone-forming agent (20).

At the doses used in the present study, SrRan did not promote analgesia in the treatment and prophylactic groups. There was no improvement in pain behavior in the animals studied, with no impact on articular mobility, motor activity, or mechanical hyperalgesia in comparison to the control group. This finding could be related to the doses used in this experimental model, which were smaller than those used in other studies on this drug, varying from 300 to 625 mg/kg (27,31). That could probably be a limitation of our study, including the time period of medication usage. Experimental improvements were obtained with higher doses (27,31) and for longer periods (15,31). This experimental model determined that additional studies examining the use of SrRan in the treatment of OA are required, particularly investigations using higher doses of this drug.
